# Strategies to increase rural maternal utilization of skilled health personnel for childbirth delivery in low- and middle-income countries: a narrative review

**DOI:** 10.1080/16549716.2022.2058170

**Published:** 2022-05-04

**Authors:** Jeanette R. Nelson, Rebekah H. Ess, Ty T. Dickerson, Lisa H. Gren, L. Scott Benson, Stephen O. Manortey, Stephen C. Alder

**Affiliations:** a Center for Business, Health, and Prosperity, David Eccles School of Business, University of Utah, Salt Lake City, Utah, USA; b Department of Family and Preventive Medicine, School of Medicine, University of Utah, Salt Lake City, Utah, USA; c Department of Paediatrics, School of Medicine, University of Utah, Salt Lake City, Utah, USA; d Department of Internal Medicine, School of Medicine, University of Utah, Salt Lake City, Utah, USA; e Ensign Global College, Kpong, Ghana; f Department of Obstetrics and Gynecology, School of Medicine; Departments of Health and Kinesiology and Health Promotion & Education, College of Health; Department of Entrepreneurship and Strategy, David Eccles School of Business; University of Utah, Salt Lake City, Utah, USA

**Keywords:** Intervention, community-based, pregnancy, maternal health services, developing country

## Abstract

**Background:**

Skilled attendance at birth is considered key to accomplishing Sustainable Development Goal (SDG) 3.1 aimed at reducing maternal mortality. Many maternal deaths can be prevented if a woman receives care by skilled health personnel. Maternal utilization of skilled health delivery services in rural areas in low- and middle-income countries is 70% compared to 90% in urban areas. Previous studies have found community-based interventions may increase rural maternal uptake of skilled health delivery services, but evidence is lacking on which strategies are most effective.

**Objective:**

To review the effectiveness of community-based strategies to increase rural maternal utilization of skilled health personnel for childbirth delivery in low-and middle-income countries.

**Methods:**

We conducted a narrative review. PubMed, CINAHL, Cochrane Library, and PsycINFO databases were searched for articles from database inception through 13 November 2019. Key search terms were pre-determined. Information was extracted on studies meeting our inclusion criteria: cluster and randomized trials, rural setting, reproductive aged women, community engagement, low- and middle-income countries. Studies were considered effective if statistically significant (p < 0.05). A narrative synthesis was conducted.

**Results:**

Ten cluster randomized trials out of 5,895 candidate citations met the inclusion criteria. Strategies included home-based visits, women’s groups, and combined approaches. Out of the ten articles, only three studies were found to significantly increase maternal uptake of skilled health personnel for delivery, and each used a different strategy. The results are inconclusive as to which strategies are most effective. Limitations of this review include heterogeneity and generalizability of studies.

**Conclusions:**

This research suggests that different strategies may be effective at improving maternal utilization of skilled health personnel for delivery in certain rural settings while ineffective in others. More research is warranted to better understand the context in which strategies may be effective and under what conditions.

## Background

It is critical to increase global efforts to improve maternal health outcomes, if the world is going to achieve the United Nations Sustainable Development Goal (SDG) maternal mortality target that calls for a global rate of less than 70 maternal deaths per 100,000 live births by 2030 [1]. While maternal mortality has decreased substantially from 2000 to 2017 from 342 to 211 deaths per 100,000 live births representing an average annual decrease of 2.9%, an average annual reduction of 6.4% is needed to meet the SDG target by 2030 [2]. The majority of maternal deaths occur in developing countries [3]. Deaths are concentrated in the two regions of sub-Saharan African (68%) and South Asia (19%), accounting for 86% of global maternal mortality [4].

Every day, more than 800 women die from complications during pregnancy and childbirth [5]. More than 70% of maternal deaths are due to direct obstetric causes: hemorrhage (27%), hypertension (14%), sepsis (11%), abortion (8%), embolism (3%), and other direct causes (10%)[6]. Most maternal deaths can be prevented if appropriate care is received by a skilled health provider [4,6]. The World Health Organization (WHO) reports that a critical intervention to reduce maternal mortality is for childbirth to be attended by skilled health personnel with access to life-saving resources [4,7]. As such, SDG 3.1 monitors the proportion of births attended by skilled health personnel (SDG 3.1.2) as a lag measure to indicate progress toward reducing the maternal mortality rate (SDG 3.1.1) [8,9]. Additionally, the benefits of utilizing skilled health personnel for delivery are extended to newborns through essential newborn care and the resulting improved survival rate [9].

Trends demonstrate that the proportion of women utilizing skilled health personnel to deliver their babies has increased across all regions in the past two decades, resulting in the global rate of births attended by skilled health personnel increasing substantially from 64% in 2000 to 83% in 2020 [10]. However, these figures conceal immense inequalities between urban and rural populations. Globally, 90% of urban births and 70% of rural births are attended by skilled health personnel [10]. Regions in sub-Saharan Africa exhibit the largest disparities, with 81% of urban births compared to 49% of rural births attended by skilled health personnel in Western and Central Africa [10]. Closing this urban-rural gap in developing countries is critical to reducing mortality rates and attaining SDG targets [10,11]. Countries have made efforts to increase maternal access to childbirth delivery services by skilled health personnel through interventions such as providing free or low-cost maternal services, increasing the number of skilled health personnel, and expanding health services to rural areas [12]. However, despite such efforts, rural women’s utilization of skilled health personnel for delivery remains suboptimal [12,13]. Traditional Birth Attendants (TBA), who lack formal healthcare training, are often preferred by rural women to assist with home birth for complex reasons including personal and cultural reasons, even though the women may recognize that TBAs are not trained to manage childbirth complications [14–16]. Thus, one of the most challenging issues in reducing maternal mortality is changing rural women’s care seeking behavior for childbirth delivery.

Community-based interventions have been demonstrated to be effective in increasing rural maternal utilization of skilled health personnel for delivery [17–19]. While reviews have been conducted on interventions that improve maternal and newborn outcomes [20–22], research is limited on the effectiveness of strategies of community-based interventions that increase maternal utilization of skilled health personnel for childbirth in rural areas of low-and middle-income countries.

Our objective was to review the effectiveness of community-based intervention strategies to increase maternal utilization of skilled health personnel for childbirth in rural areas of low- and middle-income countries.

## Methods

### Search strategy

We conducted a narrative review following a systematic approach to identify peer-reviewed, published studies evaluating community-based interventions intended to improve rural maternal utilization of skilled health personnel for childbirth delivery. This review followed the Preferred Reporting Items for Systematic Reviews and Meta-Analysis (PRISMA) guidelines [23]. We carried out an initial limited search of PubMed MEDLINE and CINAHL to identify articles on the topic. The text words contained in the titles and abstracts of relevant articles, and the index terms used to describe the articles were used to develop a full search strategy for PubMed (see Figure S1), which was adapted for each information source. A systematic search of PubMed (pubmed.gov), CINAHL Complete (Ebscohost), Cochrane Library (Wiley.com), and PsycINFO (Ebscohost) was conducted on 13 November 2019. Additionally, reference lists of included studies were hand-searched.

### Eligibility criteria

Inclusion criteria were studies of randomized and cluster randomized trials with control groups, community-based interventions, rural, and low- and middle-income countries. Population criteria included women of reproductive age, pregnant women, and others who may support pregnant women, such as men, family members, community members, and health facility staff. Outcomes included ‘skilled health personnel for delivery,’ or a related term such as ‘skilled attendant at birth’ or ‘health facility delivery’ indicating skilled health personnel presided at birth, reported as primary or secondary outcomes with an odds ratio (OR) or rate ratio (RR). No restrictions were placed on publication year or language. Studies were eligible for inclusion from the inception of databases through 13 November 2019. We excluded studies from high-income countries and urban populations and studies that did not involve community engagement or participation.

The term ‘community-based intervention’ was defined as occurring outside of a health facility and involved community members’ engagement or participation [24,25]. Identification of low- and middle-income countries was based on the 2018–2019 World Bank classification for countries [26]. Because there is no universal definition for ‘rural area,’ we accepted the authors’ classification of rural status if the study indicated the setting, population, or region was rural or remote [27,28]. If no clear population or setting was indicated, we applied the rural-region definition employed by the Organization for Economic Cooperation and Development and other institutions: ‘fewer than 150 people per square kilometre and more than 50% of the population resides in areas classified as rural communities’ [29–31]. For purposes of this review, the term ‘skilled health personnel’ refers to either the WHO’s 2004 definition of a skilled birth attendant of;

‘an accredited health professional such as a midwife, doctor or nurse who has been educated and trained to proficiency in the skills needed to manage normal (uncomplicated) pregnancies, childbirth and the immediate postnatal period, and in the identification, management and referral of complications in women and newborns’ [32,33], or the WHO’s 2018 definition in which the terminology changed to ‘skilled health personnel providing care during childbirth’ referring to health personnel who have received appropriate training and provide care in ‘an enabling environment with access to life-saving interventions and referral capacity’ [34].

In most published studies, it would be difficult to ascertain the level of birth attendant proficiency and the presence of an enabling environment referenced in the new definition, so we assumed that births attended by a skilled birth attendant or other health care provider are skilled health personnel. We also assumed that childbirths occurring in a health facility were attended by skilled health personnel.

### Study selection and data extraction

Subsequent to the search, citations were uploaded into EndNoteX8 [35], then into Covidence [36], where duplicates were removed. Two reviewers (JN, RE) independently screened titles and abstracts for eligibility and reviewed full texts of included studies. Data extraction of included studies was independently performed by two reviewers (JN, RE) on a standardized form developed to capture characteristics relevant to the studies, such as population, country, intervention, and outcome, to create a comparison table. Outcome measures included OR or RR for ‘skilled attendance at birth’ and ‘health facility delivery.’ Studies were considered effective by OR and RR cut off points for statistical significance if p < 0.05.

### Data analysis and synthesis

After reviewing extracted data in our comparison table, three predominant strategies emerged from the included studies. Studies were categorized by the predominant strategy used. These included women’s groups, home-based visits to pregnant women, or a combination of one or both of the aforementioned strategies in addition to another approach, such as involvement of men, or addressed access barriers to healthcare. We narratively synthesized relevant information to the review describing intervention, primary strategy, study population, and outcome measures. Additionally, we report on health-service strengthening activities and relevant contextual factors that authors cited that generally applied to both intervention and control groups. Due to the heterogeneity and the small number of studies, we did not conduct a meta-analysis.

## Results

**Figure 1.**
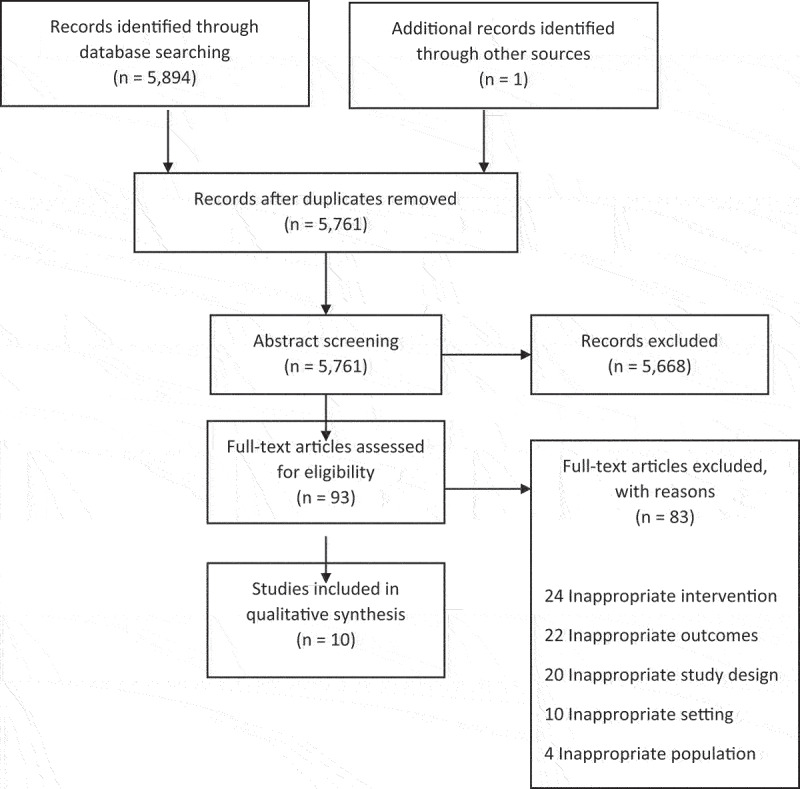
Flow diagram of study selection.

We identified 5,895 citations. After duplicates were removed, we screened 5,761 titles and abstracts (see Figure 1). We excluded 5,668 records resulting in 93 full-text articles that we retrieved and assessed for eligibility. We excluded a total of 83 articles, resulting in a total of ten studies included in the final review.

The characteristics of the ten studies are presented in Table 1; all were cluster randomized trials. Studies represented rural areas of the following low- and middle-income countries: India (3), Nepal (2), Tanzania (2), Pakistan (1), Ghana (1), and Malawi (1); seven studies represented South Asia and three represented sub-Saharan Africa. The study population of interest included women of reproductive age; one study also including husbands. Sample size ranged from 510 to 18,960 women who delivered during the intervention period. Primary strategies used by studies included home-based visits, women’s groups, and combination approaches using more than one strategy. Most authors reported conducting formative research in the study area prior to implementing the intervention [17,37–42].

**Table 1.** Characteristics of studies

First Author, Year, Country	Intervention description	Primary strategy	Study population	n	Outcome	Significance Level
Choulagai, 2017 Nepal	Five-Point Intervention of 1) increased family support for facility birth; 2) financial assistance 3) transport; 4) communication skills training for health facility staff, and 5) security for skilled birth attendants	Combination: mother’s groups and home-based visits, and other support	Cluster RCT with 36 clusters comprised of all women in the study village development, in three districts in rural Nepal, over 12 months between 2013–2014	3,844	57% increase in odds of skilled birth attendance (AOR: 1.57, 95% CI, 1.19–2.08) in intervention vs. control	p *= *<0.001
Hanson, 2015 Tanzania	Five home-based visits by trained female volunteers focused on safe motherhood and newborn care; Health service strengthening partially implemented across intervention and control areas.	Home-based visits	Cluster RCT with 132 clusters consisting of all pregnant women in intervention wards and women with live births in the three years in the control wards, in 6 districts of rural Tanzania, during July 2010-June 2013	15,373	50% increase in odds of facility delivery (AOR: 1.5, 95% CI, 1.2–2.0) in intervention vs. control	P *= *0.002
Kirkwood, 2013 Ghana	Five home-based visits facilitated by community-based surveillance volunteers focused on safe motherhood and newborn care; Health service strengthening implemented across intervention and control areas	Home-based visits	Cluster RCT with 98 clusters consisting of all pregnancies that ended in a live birth or stillbirth, in 7 districts of rural Ghana, over 12 months between 2008–2009	15,980	3% decrease rate of facility deliveries (aRR: 0.97, 95% CI, 0.81–1.14) in intervention vs. control	Not reported, not significant based on 95% CI
Kumar, 2008 India	Focused on behavior change messaging for birth preparedness and newborn care. During home-based visits, community health workers delivered a preventive package of interventions for essential newborn care or another intervention package of essential newborn care plus the use of a hypothermia indicator (ThermoSpot).	Combination of home-based visits, and community meetings	Cluster RCT with 39 clusters consisting of pregnant women, in Shivgarh, rural India, between January 2004-May 2005	2,724	41% increase rate of facility delivery with essential newborn care (aRR: 1.41, 95% CI, 0.93–2.13) in intervention vs. control	P *= *0.08
					36% increase rate in use of delivery attendant with essential newborn care (aRR: 1.36, 95% CI, 0.92–1.99) in intervention vs. control	P *= *0.11
				2,278	29% increase rate in facility delivery with essential newborn care plus ThermoSpot (aRR: 1.29, 0.83–2.02) in intervention vs. control	P *= *0.25
					38% increase rate in use of delivery attendant with essential newborn care plus ThermoSpot (aRR: 1.38, 95% CI, 0.91–2.08) in intervention vs. control	P *= *0.13
Lewycka, 2013 Malawi	Either women’s groups led by local female facilitators, or five home-based visits by volunteer peer counselors. Both interventions focused on safe motherhood and newborn care. Health service strengthening activities implemented across intervention and control areas.	Women’s groups or home-based visits	Cluster RCT with 48 clusters consisting of all women aged 10–49 years who consented to participate, in the Mchinji district in rural Malawi, between 2005–2009	18,960	27% increase in odds of Institutional delivery in woman’s groups (AOR: 1.27, 95% CI, 0.95–1.71) in intervention vs. control	Not reported, not significant based on 95% CI
					28% increase in odds of institutional delivery in volunteer peer counseling (AOR: 1.28, 95% CI, 0.82–2.00) in intervention vs. control	Not reported, not significant based on 95% CI
					22% increase in odds of birth attended by a skilled provider in women’s groups (AOR: 1.22, 95% CI, 0.91–1.65) in intervention vs. control	Not reported, not significant based on 95% CI
					21% increase in odds of birth attended by a skilled provider in volunteer peer counseling (AOR: 1.21, 95% CI, 0.79–1.86) in intervention vs. control	Not reported, not significant based on 95% CI
Manandhar, 2004 Nepal	Women’s groups facilitated by female community volunteers focused on issues of childbirth and newborn care behaviors. Health service strengthening activities implemented across intervention and control areas.	Women’s groups	Cluster RCT with 12 pairs of clusters (approx. population 7,000 per cluster) of communities in 28,931 married women aged 15–49 over 24 months from 2001–2003	3,834	212% increase in odds of births attended by government health provider; (AOR: 3.12, 95% CI, 1.62–6.03) in intervention vs. control	Not reported, but significant based on 95% CI
					253% increase in odds of births attended specifically by doctor, nurse, or midwife (AOR 3.53, 95% CI, 1.54–8.10) in intervention vs. control	Not reported, but significant based on 95% CI
					255% increase in odds of institutional deliveries (AOR: 3.55, 95% CI, 1.56–8.05) in intervention vs. control	Not reported, but significant based on 95% CI
Midhet, 2010 Pakistan	Intervention focused on safe motherhood and newborn health involving women’s groups facilitated by trained female community volunteers, traditional birth attendants trained to recognize obstetric danger signs and in clean delivery, and emergency transportation and telecom systems set up in community. Health service strengthening activities implemented across intervention and control areas.	Combination of women’s groups, men’s groups, and community engagement activities	Cluster RCT, 32 clusters of villages (approx. 2,000 total population per cluster) with ever-married women under age 50 from 1998–2002	1,858	30% increase in odds of delivery in a District Hospital (AOR: 1.3, 95% CI, 0.7–2.5) in intervention vs. control	Not reported, not significant based on 95% CI
	Same as above, but added husbands of participating women to form men’s groups facilitated by trained community male volunteer	Same as above, but added husbands of participating women	Same as above, but added husbands of participating women	1,725	30% increase in odds of delivery in a District Hospital (AOR: 1.3, 95% CI, 0.6–2.7) in intervention vs. control	Not reported, not significant based on 95% CI
Penfold, 2014 Tanzania	Five home-based visits by trained community volunteers aimed at educating women on safe motherhood and newborn care.Health service strengthening partially implemented across intervention and control areas.	Home-based visits	Cluster RCT, 57 pairs of clusters (approx. total population 1.2 mill.) with childbearing women aged 13–49 from 2010–2011	510	40% increase in odds of birth attended by skilled birth attendant (AOR: 1.4, 95% CI, 0.9–2.3) in intervention vs. control	P *= *0.16
				609	40% increase in odds of health facility delivery (AOR: 1.4, 95% CI, 0.9–2.3) in intervention vs. control	P *= *0.14
Tripathy, 2010 India	Women’s groups facilitated by trained local female community worker consisting of monthly sessions on maternal and newborn health. Health service strengthening activities implemented across intervention and control areas.	Women’s groups	Cluster RCT, 36 clusters (approx. population 228,186) with women of reproductive age (15–49 years) from 2005–2008 over 3 years	18,335	19% decrease in odds of birth attended by formal provider (AOR: 0.81, 95% CI, 0.50–1.31) in intervention vs. control, for years 1–3	Not reported but not significant based on 95% confidence interval
					11% decrease in odds of institutional deliveries (AOR: 0.89, 95% CI, 0.51–1.53) in intervention vs. control, for years 1–3	Not reported but not significant based on 95% CI
Tripathy, 2016 India	Women’s groups facilitated by village-based, trained female government-approved Accredited Social Health Activists (ASHAs) consisting of monthly sessions on maternal and newborn health. Health service strengthening activities implemented across intervention and control areas.	Women’s groups	Cluster RCT, 30 clusters (approx. population 156,519) with women of reproductive age (15–49 years) from 2010–2013 over 31 months	7,100	Model 1: 23% increase in odds of health facility birth (AOR: 1.23, 95% CI, 0.58–2.60) in intervention vs. control	Not reported but not significant based on 95% CI
					Model 2: 20% increase in odds of health facility birth (AOR: 1.20, 95% CI, 0.81–1.78)	Not reported but not significant based on 95% CI

AOR: adjusted odds ratio; aRR: adjusted risk ratio.

Outcomes of interest for this review included ‘skilled health personnel delivery’ and ‘health facility delivery.’ One article reported the outcome of interest as a primary outcome [17], while the other nine reported it as a secondary outcome [37–45]. Authors of eight of the ten studies reported implementing health service strengthening activities across both intervention and comparison areas [37,38,40–45].

Three studies were reported as having statistical significance (p *< *0.05, 95% confidence intervals, or CI)s for increasing maternal utilization of skilled health personnel for delivery or health facility delivery [17,37,40]. In comparison, though not statistically significant, five studies trended in the direction of increased maternal utilization of skilled delivery care compared with the comparison group [39,41–43,45].

### Interventions using home-based visits as a primary strategy

Researchers in four studies reported implementing interventions using home-based visits to pregnant women by community health workers as a primary strategy [37,38,42,43] (see Table 1). During interventions, women receive health education and counselling in their home from trained community health workers on safe motherhood and newborn care practices, including the importance of giving birth utilizing skilled health personnel. Each of the studies employed five home-based visits, although the timing of visits varied, ranging from one to three visits during pregnancy with the remainder following birth. Three of the studies reported increases in the utilization of ‘skilled health personnel delivery’ or ‘health facility delivery’ [37,42,43]. However, only the Hanson study in Tanzania (OR: 1.5, CI: 1.2–2.0, p *= *0.002) was statistically significant based on reported p values or 95% CI for women exposed to the intervention compared to the control group [37].

### Interventions using women’s groups as a primary strategy

Four articles reported results on interventions using women’s groups [40,43–45] as shown in Table 1. Women’s groups are generally facilitated by a trained female facilitator using a participatory approach. Regular meetings often held monthly, employed a four-phase structure that included identifying and prioritizing maternal and newborn issues, planning strategies to address identified issues, implementing prioritized strategies, and assessing implemented strategies [40,43–45]. Additional meetings are generally held with community members to enlist support [40,43–45].

Three of the articles reported increases [40,43,45] in the utilization of ‘skilled health personnel delivery’ or ‘health facility delivery’ between intervention and comparison groups. However, only the Manandhar study in Nepal was statistically significant based on reported p values or 95% CI, reporting increased odds of births attended by government health provider (OR: 3.12, 95% CI: 1.62–6.03), births attended specifically by a doctor, nurse, or midwife (OR 3.53, 95% CI: 1.54–8.10), and health facility deliveries (OR: 3.55, 95% CI: 1.56–8.05) for intervention clusters compared with control clusters [40].

### Interventions using combinations of strategies

Three articles reported interventions employing more than one strategy [17,39,41], as shown in Table 1. The Choulagai study in Nepal implemented a five-point intervention using multiple strategies, including mother’s groups, home visits, health facility staff training, and community support [17]. The intervention involved 1) increased family support for childbirth in a health facility, 2) financial assistance for women seeking delivery at a health facility, 3) transportation at the time of childbirth, 4) two-day training for health facility staff on communication skills, positive attitude to support women and their families at the time of childbirth, and 5) increased night-time security for skilled health attendants [17]. The Choulagai article reported increased odds in the utilization of skilled health personnel at birth for intervention clusters compared to control clusters, showing statistical significance at p < 0.05 (OR: 1.57, 95% CI: 1.19–2.08, p *= *<0.001) [17].

The remaining two studies, while not statistically significant, trended toward improving the utilization of skilled health personnel for childbirth. The Kumar study in India used a combination of strategies focused on behavior change messaging for birth preparedness and newborn care that included a combination of women’s groups, home visits, community meetings, and stakeholder meetings [39]. Similarly, the Midhet study in Pakistan was focused on behavior change, employing women’s and men’s groups’ strategies, implementing transportation and telecommunications systems, and training traditional birth attendants to recognize obstetric and newborn danger signs [41]. Unlike other women’s groups represented in this review, the Midhet intervention used an educational program with audiocassettes and pictorial books focused on safe motherhood and newborn health, called information and education for empowerment and change [41]. The intervention was delivered to groups of women and separately to groups of participating husbands [41].

### Health service strengthening

Most studies reported health service strengthening activities implemented across both intervention and comparison areas [37,38,40–45], although researchers in two of the studies reported only partial implementation across both intervention and control sites due to financial constraints [37,42]. Alternately, researchers in the Choulagai study in Nepal reported health service strengthening activities as part of the intervention in the experimental group only [17]. In contrast, the Kumar study in India did not report specific health service strengthening activities, reporting that health system workers in the intervention area were engaged at the community level only, as part of intervention stakeholder meetings that included traditional and unqualified providers, to ensure consistent messaging of targeted behavior change practices [39].

The most common health service strengthening activity included appropriate level training in obstetric and newborn care for health care workers [38,40,41,43], community health volunteers [40], and although not part of the formal health system, traditional birth attendants [38–41]. Other types of instructional activities reported included communication-skills training for health staff and creating a women-friendly environment [17], workshops with health care providers for appreciative inquiry [44], and meetings with government officials and hospital management to advise on the provision of appropriate care in health facilities for mothers and newborns [45]. Additional activities reported included facility-based quality of care [37,42], equipping health facilities with obstetric and newborn care equipment [40,41,43], equipping government trained community health volunteers with educational materials and newborn kits [40], increased night-time security for skilled health personnel [17], forming health committees [44], and conducting meetings with health committees concerning maternal and newborn health rights and entitlement rights [44,45].

### Additional contextual factors

All interventions occurred on top of existing standard health services and programs and any health strengthening activities implemented across both intervention and control areas. Two studies implementing interventions working with women’s groups as primary strategies reported that government-trained community health workers already functioned in the study area conducting home-based visits to improve maternal and newborn health [40,44]. Furthermore, researchers in one study implementing an intervention using home-based visits as a primary strategy reported that other programs from various organizations concurrently operated in the study area encouraging facility delivery, including a national antenatal program, other facility-based quality improvement programs, and other home-based visit programs [37]. Researchers in the same study noted the socioeconomic context, reporting that the intervention occurred in an environment that was rapidly transforming, in which there were substantial increases in the availability of motorcycle transportation and mobile phones in the entire study area [37].

## Discussion

Our findings indicate there is insufficient evidence to determine which types of community-based strategies are most effective at increasing rural maternal utilization of skilled health personnel for delivery; this is due to the limited number of studies demonstrating statistical significance. Of the ten studies, three showed statistically significant increases in the outcome of ‘skilled health personnel delivery’ or ‘health facility delivery’ between the intervention and the control groups, each employing a different strategy [17,37,40]. The Manandhar intervention used women’s groups as a primary strategy and additionally reported substantial inputs across intervention and control areas that included obstetric and newborn care training, equipping health facilities, and providing supplies to government-trained community health volunteers performing home-based visits in the study area [40]. In contrast, the Choulagai study used a combination of strategies involving mother’s groups, home-based visits, community engagement, and health staff training to deliver a five-point intervention but did not implement any health system strengthening activities in the control area [17]. Alternately, the Hanson study used home-based visits as a primary strategy and additionally reported partial implementation of a facility-based quality improvement program across intervention and control areas [37].

Although the results of this review are inconclusive, it suggests some important findings. First, different strategies may be effective in some settings while not in others, suggesting that context of existing health services and community factors is important. Second, interventions may need to address multiple factors employing different types of strategies, including health system strengthening activities, depending on baseline factors that may limit the success of interventions, such as unavailability of health staff.

Additionally, while it is revealing that most studies in our review did not show a statistically significant impact on the utilization of skilled health personnel for delivery, neither did a particular strategy prove more effective. Several possible explanations for this exist. Maternal behavior change may be challenging, despite the implementation of various strategies aimed at educating mothers on maternal and newborn health practices; this may be due to a multitude of factors, including established cultural beliefs and behaviors [46]. Other personal and structural barriers may also influence care-seeking [46] that may be challenging for interventions to address adequately, regardless of the strategy used. Social, cultural, financial, and geographic barriers are known to influence maternal care-seeking [46]. Indeed, sociocultural factors of long-held traditions and community practices that limit or affect maternal decision-making and care seeking behavior have been identified as barriers to utilization of skilled health delivery services in both sub-Saharan Africa [16,47–49] and South Asia [50,51]. Additionally, health systems themselves may present additional barriers, such as poor care quality and resource limitations [52,53]. There are many other possible explanations, including quality of implementation [54], differences in the socioeconomic and educational status of the population studied [55], local economic conditions [55], and program leadership [54].

Other studies corroborate our findings. A 2019 review on community health educational interventions reported a nonsignificant impact of one-to-one counselling, group counselling, or combined group and one to one counselling on increasing skilled attendance at birth [56]. Similarly, a review on participatory women’s groups showed that only one study out of a total of seven showed an increase in institutional deliveries, and in meta-analysis found no impact on maternal mortality [57]. Our review differs in that it is the first review to compare different types of intervention strategies aimed at increasing rural maternal utilization of skilled health personnel for delivery.

The strength of this study is that a standard, systematic process was followed. Additionally, to our knowledge, this is the first study reviewing community-based strategies in rural settings aimed at increasing maternal utilization of skilled health personnel for delivery. Limitations of this review include the heterogeneity of studies, making it difficult to compare studies directly, and generalizability of studies. Interventions found to be effective in one rural setting may not be so in other rural areas.

### Implications and recommendations

As the world works towards achieving SDG 3 maternal health targets by 2030, there is an urgent need for more high-quality studies of community-based interventions to determine which strategies are most effective in which contexts and under which conditions to increase rural maternal utilization of skilled health delivery services. Particular focus is needed in sub-Saharan Africa where few high-quality studies on this topic have been conducted, yet the Region suffers the highest burden of maternal deaths and has the largest disparity between urban and rural births attended by skilled health personnel.

The sociocultural conditions under which care seeking occurs that differ between contexts should be addressed as this may influence maternal uptake of skilled health services more profoundly than cost and access issues. It is critical that sociocultural factors limiting women’s decision-making and autonomy to utilize skilled health services be identified and overcome. It is equally important to recognize that women may prefer customary community practices such as the use of TBAs, and to find acceptable ways to address such factors. Additionally, health system factors may need to be addressed. Further research may benefit from using other methodologies, or by applying a theoretical behavioral model to understand factors influencing maternal care seeking to inform development of future intentions.

Future policies and interventions may require a multifactorial approach to address contextual factors and conditions that differ between societies. Policies should be implemented to ensure women are able to overcome geographical, financial, health systems, and sociocultural factors to receive skilled health services when giving birth. In addition to policies that improve access to care, it is crucial that policies be established that empower women and increase women’s autonomy, so they have the decision-making power to seek skilled health services. While it is critical to identify and implement strategies to increase rural maternal utilization of skilled health personnel for delivery to reduce maternal mortality, it is equally important to recognize and address root causes related to this important issue. It is well known that rural women with low education and socioeconomic levels are disproportionately affected, accessing skilled health services for childbirth at lower rates compared with more advantaged women [58]. For policies and interventions to be successful, it is essential that they are pro-equitable, so the least advantaged are able to benefit.

## Conclusion

Research is limited regarding effective strategies for increasing rural maternal utilization of skilled health personnel for delivery. This research suggests that different strategies may be effective in some rural settings while having limited impact in others. More high-quality research is needed to better understand which strategies are effective under which conditions, and researchers should consider the sociocultural context. Additionally, further research may benefit from other research methodologies or from applying a theoretical health behavior model to better understand rural maternal care-seeking behavior, and to inform development of future interventions.

## Supplementary Material

Supplemental Material

## Paper context

Increased action is required if the world is to reduce global maternal mortality rates to the UN SDG target of fewer than 70 deaths per 100,000 live births by 2030. The majority of maternal deaths can be prevented if a woman utilizes health services from skilled health personnel. The maternal uptake of skilled health deliveries is particularly poor in rural areas within low- and middle-income countries. This review presents a narrative synthesis of community-based strategies aimed at increasing rural deliveries attended by skilled health personnel.
