# Supplementary material for: Strategies to increase rural maternal utilization of skilled health personnel for childbirth delivery in low- and middle-income countries: a narrative review
Source: Glob Health Action. 2022 May 4;15(1):2058170. doi: 10.1080/16549716.2022.2058170 (PMC9090426; doi:10.1080/16549716.2022.2058170)
Supplement: Supplemental Material [file ZGHA_A_2058170_SM1208.docx]

**Supplementary Information**

**Figure S1. Search Strategy for Pubmed**

(((((((((((("Parturition"[Mesh:NoExp] OR "Birth Setting"[Mesh:NoExp] OR "Home Childbirth"[Mesh:NoExp] OR "Natural Childbirth"[Mesh:NoExp]))) OR ((birth[Text Word] OR births[Text Word] OR "child birth*"[Text Word] OR child-birth*[Text Word] OR childbirth*[Text Word] OR "home birth*"[Text Word] OR home-birth*[Text Word] OR parturition*[Text Word] OR puerperal[tw] OR puerperium[tw] OR postpartum[tw])))))) OR ("Pregnancy"[Mesh] OR "Pregnancy Complications"[Mesh])) OR "Delivery, Obstetric"[Mesh]) OR ("Maternal Death"[Mesh] OR "Maternal Health"[Mesh] OR "Maternal Welfare"[Mesh] OR "Maternal Mortality"[Mesh] OR "Maternal-Child Health Centers"[Mesh] OR "Maternal Health Services"[Mesh] OR "Perinatal Care"[Mesh] OR "Postnatal Care"[Mesh] OR "Preconception Care"[Mesh] OR "Prenatal Care"[Mesh] OR "maternal death*"[Text Word] OR "maternal mortality"[Text Word] OR "maternal health"[Text Word] OR "maternal welfare"[Text Word] OR "perinatal care"[Text Word] OR "postnatal care"[Text Word] OR "preconception care"[Text Word] OR "prenatal care"[Text Word] OR "antenatal care"[Text Word]) AND ((skilled[Text Word] AND attendan*[Text Word]) OR "skilled health personnel"[Text Word] ))

OR

(((((("Health Services"[Mesh:noExp] OR "Community Health Services"[Mesh] OR "Reproductive Health Services"[Mesh] OR "Reproductive Health"[Mesh] OR "Women's Health Services"[Mesh])) AND ((community-based[Text Word] OR "community based"[Text Word] OR communities-based[Text Word] OR "communities based"[Text Word] OR "community participation"[Text Word]))) AND (((((((("Parturition"[Mesh:NoExp] OR "Birth Setting"[Mesh:NoExp] OR "Home Childbirth"[Mesh:NoExp] OR "Natural Childbirth"[Mesh:NoExp]))) OR ((birth[Text Word] OR births[Text Word] OR "child birth*"[Text Word] OR child-birth*[Text Word] OR childbirth*[Text Word] OR "home birth*"[Text Word] OR home-birth*[Text Word] OR partuition*[Text Word] OR "water birth*"[Text Word] OR "lamaze technique*"[Text Word] OR puerperal[tw] OR puerperium[tw] OR postpartum[tw])))))) OR ("Maternal Death"[Mesh] OR "Maternal Health"[Mesh] OR "Maternal Welfare"[Mesh] OR "Maternal Mortality"[Mesh] OR "Maternal-Child Health Centers"[Mesh] OR "Maternal Health Services"[Mesh] OR "Perinatal Care"[Mesh] OR "Postnatal Care"[Mesh] OR "Preconception Care"[Mesh] OR "Prenatal Care"[Mesh] OR "maternal death*"[Text Word] OR "maternal mortality"[Text Word] OR "maternal health"[Text Word] OR "maternal welfare"[Text Word] OR "perinatal care"[Text Word] OR "postnatal care"[Text Word] OR "preconception care"[Text Word] OR "prenatal care"[Text Word] OR "antenatal care"[Text Word])))) AND (("Developing Countries"[Mesh] OR "developing countries"[Text Word] OR "less developed countries"[Text Word] OR "least developed countries"[Text Word] OR "third world countries"[Text Word] OR "under developed countries"[Text Word] OR "developing nation*"[Text Word] OR "less developed nation*"[Text Word] OR "Low- and middle-income countries"[Text Word] OR "Low and middle income countries"[Text Word] OR lmic[tiab] OR lmics[tiab]))))

OR

((("Maternal Death"[Mesh] OR "Maternal Health"[Mesh] OR "Maternal Welfare"[Mesh] OR "Maternal Mortality"[Mesh] OR "Maternal-Child Health Centers"[Mesh] OR "Maternal Health Services"[Mesh] OR "Perinatal Care"[Mesh] OR "Postnatal Care"[Mesh] OR "Preconception Care"[Mesh] OR "Prenatal Care"[Mesh] OR "maternal death*"[Text Word] OR "maternal mortality"[Text Word] OR "maternal health"[Text Word] OR "maternal welfare"[Text Word] OR "perinatal care"[Text Word] OR "postnatal care"[Text Word] OR "preconception care"[Text Word] OR "prenatal care"[Text Word] OR "antenatal care"[Text Word])) AND (("Developing Countries"[Mesh] OR "developing countries"[Text Word] OR "less developed countries"[Text Word] OR "least developed countries"[Text Word] OR "third world countries"[Text Word] OR "under developed countries"[Text Word] OR "developing nation*"[Text Word] OR "less developed nation*"[Text Word] OR "Low- and middle-income countries"[Text Word] OR "Low and middle income countries"[Text Word] OR lmic[tiab] OR lmics[tiab]) OR "Rural Health"[Mesh] OR "Rural Population"[Mesh] OR rural[tw]) AND ("Outcome Assessment (Health Care)"[Mesh] OR outcome[ti] OR outcomes[ti] or intervention[ti] OR interventions[ti] OR evaluation[ti] OR trial[ti] OR project[ti] OR program[ti] OR programme[ti] OR pilot[ti] OR study[ti])))
